# When actions do not match aspirations - comparison of the European Union policy claims against what has been negotiated for health services, trade and investment

**DOI:** 10.1186/s12992-021-00739-8

**Published:** 2021-08-30

**Authors:** Meri Koivusalo, Noora Heinonen, Liina-Kaisa Tynkkynen

**Affiliations:** Tampere University, Tampere, Finland

**Keywords:** Health systems, Trade, Globalization, European Union, Health policy

## Abstract

**Background:**

Obligations arising from trade and investment agreements can affect how governments can regulate and organise health systems. The European Union has made explicit statements of safeguarding policy space for health systems. We assessed to what extent health systems were safeguarded in trade negotiations using the European Union (EU) negotiation proposals for the Transatlantic Trade and Investment Partnership (TTIP) and the negotiated agreement for the EU-Canada Comprehensive Economic and Trade Agreement (CETA).

**Methods:**

We assessed if and to what extent the European Union policy assurances were upheld in trade negotiations. Our assessment was made using three process tracing informed tests. The tests examined: i) what was covered in negotiation proposals of services and investment chapters, ii) if treatment of health services differed from treatment of another category of services (audiovisual services) with similar EU Treaty considerations, and iii) if other means of general exceptions, declarations or emphases on right to regulate could have resulted in the same outcome.

**Results:**

Our analysis shows that the European Union had sought to secure policy space for publicly funded health services for services chapter, but not for investment and investment protection chapters. In comparison to audiovisual services, exceptions for health services fall short from those on audiovisual services. There is little evidence that the same outcome could have been achieved using other avenues.

**Conclusions:**

The European Union has not achieved its own assurances of protection of regulatory policy space for health services in trade negotiations. The European Union trade negotiation priorities need to change to ensure that its negotiation practices comply with its own assurances for health services and sustainable financing of health systems.

## Background

Trade agreements affect public health and health services regulation. We already have research and analysis in relation to tobacco control [1–6], alcohol [7, 8], food [9, 10], and access to and prices of medicines [11, 12]. Health services have been analysed in relation to World Trade Organisation General Agreement on Trade in Services (GATS) [13–15]. We have prior research on how new generation trade agreements influence national and local policy space for health promotion and health services regulation [15, 16]. We thus know that trade negotiations can have impact on how governments regulate for health protection, health promotion, equity and access to health care, and sustainability of financing of health services.

European Union has made formal promises and policy statements in relation to health services. European Union formal policy stance on trade and investment is that [17]: “EU trade agreements do not and will not prevent governments, at any level, from providing, supporting or regulating services in areas such as water, education, health, and social services, nor will they prevent policy changes regarding the financing or organisation of these services”. This policy stance is from the key EU trade policy document in 2015 and echoes what was emphasised in a joint statement on public services by EU and US in 2015 for TTIP negotiations [18].

This article is based on assumption that i) trade negotiations on services and investment can affect **how** governments regulate and subsidise services and investment, and ii) that the greatest policy space for governments is achieved, when services are not included under trade and investment agreements. Governments have been reluctant to include health services under trade agreements due to a variety of reasons, including values on which health systems function or need to interfere with markets, for example, to seek cost-containment or equity. Governments need the scope to regulate and subsidize health services provision on the ground of public and health policy priorities. Regulatory policy space is important, for example, to ensure adequate risk-pooling for health systems, to limit fragmentation of service provision, to secure sustainable services in remote areas, to ensure access to data and full oversight on safety and quality, and to ensure financial sustainability of the health system. From the perspective of regulatory policy space, excluding health systems as whole from trade and investment agreements would give most predictability and policy space for public policies.

The European Union statement reflects the long-term concerns over services trade and health. The specific role of health, social, educational, audiovisual and cultural services is embedded in the reservations in the carve out in the Treaty of Nice. Negotiations on investment and investment protection in the EU-Canada Comprehensive Economic Partnership Agreement (CETA) and the proposed EU-US Transatlantic Trade and Investment Partnership (TTIP) represent new areas for FTAs and thus differ from ordinary bilateral trade agreements.

The European Union functions under multilevel governance. The European Commission has exclusive competence on common commercial policy as set in Article 3 in the Treaty on the Functioning of the European Union [19] (Table 1). This means that the European Commission is negotiating trade agreements on behalf of the Member States. Exclusive competence on trade negotiations is moderated by practice of negotiating agreements in consultation with the European Union Member States as well as recognising, where negotiators need to take into account the broader European Union Treaty exceptions empowering Member State competence as set in paragraph 4 in Article 207 TFEU on common commercial policy [19]. The Treaty of Nice expanded the European Union competence in services trade, but it still included a specific exception requiring shared competence with respect to health, social and education services. This forms the background for the current Article 207 (4) TFEU (Table 2) in the Lisbon Treaty. The same Article includes reservations with respect to cultural and audiovisual services (Table 2). However, this “carve out” in the Lisbon Treaty is a last resort measure and politically demanding to raise as it requires that a MS challenges negotiation results and makes the case how the negotiated agreement is a threat to financial sustainability of health services.

**Table 1:** Union competence in trade policy (TFEU, 2012)

Article 3 1. The Union shall have exclusive competence in the following areas: (a) customs union; (b) the establishing of the competition rules necessary for the functioning of the internal market; (c) monetary policy for the Member States whose currency is the euro; (d) the conservation of marine biological resources under the common fisheries policy; (e) common commercial policy. 2. The Union shall also have exclusive competence for the conclusion of an international agreement when its conclusion is provided for in a legislative act of the Union or is necessary to enable the Union to exercise its internal competence, or in so far as its conclusion may affect common rules or alter their scope.

**Table 2:** Article 207 paragraph 4 in Treaty of Functioning of the European Union (TFEU 2012)

4. For the negotiation and conclusion of the agreements referred to in paragraph 3, the Council shall act by a qualified majority. For the negotiation and conclusion of agreements in the fields of trade in services and the commercial aspects of intellectual property, as well as foreign direct investment, the Council shall act unanimously where such agreements include provisions for which unanimity is required for the adoption of internal rules. The Council shall also act unanimously for the negotiation and conclusion of agreements: (a) in the field of trade in cultural and audiovisual services, where these agreements risk prejudicing the Union’s cultural and linguistic diversity; (b) in the field of trade in social, education and health services, where these agreements risk seriously disturbing the national organisation of such services and prejudicing the responsibility of Member States to deliver them.	

In trade negotiations the World Trade Organisation agreements are considered as the floor for negotiations on bilateral or plurilateral basis, which seek to go deeper and expand negotiations further than WTO agreements. One example of plurilateral agreements is the Trade in Services Agreement (TiSA), which initially dominated services negotiations in the early 2000s, before the shift to more comprehensive trade and investment agreements emerged. The new generation trade agreements are usually “mixed” agreements including chapters, which can be concluded by the European Union, but as well chapters, which need to be ratified by the 27 Member States. In comparison to trade in goods and tariffs, services and investment chapters extend more to national policymaking at different levels of governance.

Policy space can be defined as: “The freedom, scope, and mechanisms that governments have to choose, design, and implement public policies to fulfil their aims.” [20]. Policy space covers the scope for health services regulation as well as policy measures for cost-containment in health systems. In services policy space can become an issue, when health services are publicly financed, but provided by private non-profit and commercial providers. Governments are more likely to breach trade obligations when they limit markets or restrict provision or profits of commercial health services providers. Trade agreements can restrict national governments’ policy space both in terms of how health systems are organised and regulated as well as how public health standards are set. It is thus important to understand how the EU negotiates the agreements.

Articulation of national priorities, emerging populist regimes, and emergence of the covid-19 pandemic has been reflected in some stagnation of trade negotiations. However, it can be expected that trade policies and negotiation of trade agreements will emerge as the remedy for ailing economies in the aftermath of covid-19 crisis measures. This articulation is also present in the European Union policy documents and expectations of benefits from negotiated trade agreements for economic recovery [21, 22].

Trade-related health impacts are usually implicitly or explicitly, based on expectations of welfare impacts from economic growth as result of more predictable and non-discriminatory regulatory context [23, 24]. While health services are increasingly considered as tradable services, they have not been at the core of trade policy debates in services trade [13]. However, what is perceived as “improvement” in liberalisation of services, regulatory cooperation or intellectual property rights by trade negotiators and export industries, can be a “problem” for national and local health policy priorities, and obligations to ensure a high level of health protection in all policies. This the case, for example, if obligations arising from trade agreements undermine scope for cross-subsidization within health systems, increase prices of medicines, or make harder to impose health protection-related legislation.

The European Union has engaged with trade negotiations on so called “new generation trade agreements” or “mega regional deals”, such as the EU-Canada Comprehensive trade agreement (CETA) and the EU-US Transatlantic trade and investment partnership (TTIP) [25, 26]. The term “new generation” trade agreement is used here to indicate a shift towards a more comprehensive reach from tariffs to so called “beyond-the-border” issues and rules-related negotiations. Young [27] has emphasised how the TTIP negotiations differed from traditional trade negotiations due to mutual interests in businesses across Atlantic, but opposition arising from consumer, environmental and labour groups. In other words, key differences were not between different business interests, but between business and other policy interests. While the TTIP negotiations have been articulated as a “game changer” in trade policy [28, 29], we include in our analysis also CETA due to similarities in key chapters on services and investment. Canada can also be seen as an entry point to North-American trade talks and transatlantic cooperation. Negotiations on CETA implied negotiating in North American Free Trade Agreement (NAFTA) context, which would contribute towards forthcoming TTIP negotiations. Analysis of “new generation” comprehensive agreements and agreements between high-income countries is relevant as these “new generation” agreements have expanded the scope of trade agreements to new areas, formalised and widened services commitments, and opened new chapters for negotiations, such as investment and investment protection. Another reason is that bilateral agreements have relied on relatively similar claims, priorities, and chapters [30]. In this analysis we have thus focused on path-breaking agreements, where change has been sought, rather than examining a high number of similar bilateral agreements. WTO agreements form the minimum requirements for trade negotiations, which are progressive and build on prior negotiated trade agreements. CETA and TTIP expanded the negotiation agenda in several areas. As result of negative listing new categories of services previously not included under GATS were included, new chapters on rules-based negotiations in services were taken further, chapters on investment and investment protection were included as well as on regulatory cooperation. In this article we focus on services and investment, understanding that similar analysis could and should be made in relation to other negotiated chapters.

New generation trade agreements include investment and investment protection. Investment protection is a concern for the EU Member States (MS) as it applies to all services, including health services. We have already European investment protection cases on health services and health insurance [31, 32]. For example, in a case against Slovakia, insurance company Achmea made a claim for compensation after Slovakia legislated against profits under its publicly funded health insurance system against the intra-EU bilateral agreement [31]. The claim was judged against Slovakia and included an explicit statement that if Slovakia did not wish to include health services under investment protection, it should not have included health services under investment protection [31]. This would support our emphasis on exclusion of health services. Investment protection has also been raised as a competence issue between the EU and its Member States. The European Court of Justice opinion on competence proposed that free trade negotiations would negotiate investment protection separately from other trade negotiations [33].

## Methods

Our analysis has its focus on negotiation documents as these provide the “hard data” on what was negotiated. In our analysis we interpret that as a minimum the EU formal commitment should imply that the EU trade negotiation positions on services and investment should not affect provision or limit regulation of these services. In the light of the investment arbitration case against Slovakia, this would require exclusion of such services from trade in services and investment negotiations.

Our analysis examined if European Commission has “walked the talk”, i.e. if public policy stances have become realised in safeguarding health in negotiation proposals and negotiated texts on the ground of three tests: i) in relation to services and investment chapters and schedules for services, ii) in comparison to measures concerning other “like” service (audiovisual services) -related commitment, and iii) with respect to relevance of claims of general principles and exception clauses as alternative avenues for a similar outcome.

We used comparative textual and legal analysis following broadly process tracing and design of the three tests/thematic emphases [34]. We used formal publicly available trade policy stances, trade negotiation mandates, agreed texts and the EU offers to assess to what extent claims of safeguarding policy space for health services remained valid. Our interest was thus not only on what was negotiated to be included in trade agreements, but as well on the European Commission’s own negotiation offers, which it is free to make and propose. We used audiovisual services as a reference point as a comparable other services-related commitment under EU Lisbon Treaty. Audiovisual services were included under Lisbon Treaty Article 207(4) with similar emphasis as health and social services. The test sought to examine priorities and potential. In audiovisual services concerns related, for example, to the position of public monopolies as well as how national film industries are supported and maintained. These are comparable to health services concerns related to publicly funded and solidarity-based health systems. The comparison was thus between how health and another service category were treated in negotiations. Finally, to avoid ignoring potential impact of general exceptions and principles, which could solve the problem irrespective of negotiated texts, we examined potential for using general “public services” clauses or other avenues to achieve the same outcome.

## Results

### The test on services and investment negotiations

In practice market access has often been open for services trade between high-income countries for services not formally included in trade agreements, such as health services. This gives governments freedom to manage, subsidise and organise services and reserves the right to return more easily to public services from outsourced services. In terms of the first “test” our focus was on services and investment chapters negotiations and to what extent negotiation texts had excluded or kept health services outside trade or investment obligations. Negotiations consist of text negotiations concerning agreed text and negotiation of sectors and services, which are included or excluded from the agreement in annexes.

A key feature of new generation trade agreements is a move towards negative listing. In contrast to the WTO GATS-agreement, negative listing includes all services under trade agreement, except those which are specifically excluded. Negative listing and using existing legislation (standstill) as the reference in the CETA and the TTIP expand negotiations and make it harder for governments to exclude services. Governments need to define and articulate reservations and exceptions to keep these outside negotiations. Furthermore, a ratchet mechanism will include a service automatically as part of trade agreement as legislation becomes liberalised [16, 35]. Thus in contrast to listing of services to be included to the agreement (i.e. positive listing as in GATS), the negotiations in the CETA and the TTIP have moved towards “North American Free Trade Agreement (NAFTA)“– style of negotiations. In the CETA [36] negative listing of reservations in the annexes have been further divided to three categories of listing, with i) a list of services with “incompatible” regulation, which will be automatically included under the agreement obligations, if the law or regulations are changed (ratchet), ii) the list which allows policy space for future regulation, which is not compatible with the trade agreement obligations, and iii) the third very short list including fully removed services.

Trade agreement negotiations using negative listing and “ratchet” provisions are biased towards liberalisation as governments cannot predict all future regulatory needs or to anticipate what to keep outside of the agreements and negotiations. As result of change in negotiation aims to include all services through negative listing, liberalisation and market-based regulation has now become the new “normal” as reference. The Ministries of Health are – if they are asked – required to articulate and show evidence for exceptions from this rule to the Ministries of Trade within Member States and further to the European Commission.

While the European Commission trade negotiation aims seek to expand from what is included under previous trade agreements, there are exceptions. In the case of services negotiations, the European Commission has exclusion of “any publicly funded health services” in CETA, which has expanded the scope of the exception from what have been set under the WTO agreements by some countries in the GATS [37]. However, this restriction is not applied to all public services. It thus represents a clear response to Member State concerns and European Parliament resolutions seeking to exclude publicly funded health and social services. On the other hand, “any publicly funded health services” can be considered as an alternative to the broader prior use of services of general economic interest or public utilities exception [24]. In this respect EU has prioritised health and social services in comparison to other public services.

Negative listing has introduced more overarching general obligations for services. The structure of negotiations has also become changed to include separate sections in new chapters in CETA [36]. This can be seen in trade documents, for example, in separate chapters on domestic regulation, mutual recognition of qualifications, investment protection, regulatory cooperation, and standardisation under technical barriers to trade [36, 38, 39]. Domestic regulation obligations apply to how governments can set technical standards and licensing and could push regulatory measures towards least market restrictive options [23, 40]. This would affect how and on what ground domestic regulations can be set and, for example, if and on what grounds governments may limit persons’ choice of a service provider for health service or insurance, or how licensing of eligible providers can be done [40].

The most prominent questions relate to investment liberalisation and protection. New generation trade agreements have separate investment chapters, which focus on both investment liberalisation and protection. While the Member States have often excluded health services from commercial presence (mode 3) under services chapter in their listing of services in the annexes to the agreement text, this is not necessarily followed in investment chapters, where all services and sectors can be included. One example of utilising new chapters can be seen in how the European Commission has suddenly divided national treatment in investment into establishment and national treatment. Establishment now corresponds to what Member States have excluded in services chapter (Annex II exclusions). However, this is now separated from national treatment of investments already in a country. This is evident in the national treatment provision as proposed in European Unions’ own negotiation proposals for TTIP, which has been split into two paragraphs [38] (Table 3). The second paragraph is, however, not included in the list of investment liberalisation exclusions under Article 2–7 on reservations and exceptions (Table 3). It is thus subject not only to full national treatment obligations, but as well to the right of foreign investors to raise claims under investment arbitration [38]. As market access for health services (i.e. establishment) remains open in most EU Member States, what really matters for regulation and policymaking are requirements for national treatment, which can affect more government regulatory measures, permissions and standards. However, investment obligations now apply to health services in the European Unions’ own proposal in the TTIP negotiations [38]. Furthermore, this is done even though several Member States have explicitly excluded commercial presence (mode 3) in health services in their national listing of reservations/exclusions in Annex II.

**Table 3:** EU TTIP proposal investment liberalization chapter

National Treatment provisions in investment chapter	
Article 2–3 National Treatment 1. Each Party shall accord to investors of the other Party and to their investments, as regards the establishment of an enterprise in its territory, treatment no less favourable than the treatment it accords, in like situations, to its own investors and their investments. 2. Each Party shall accord to investors of the other Party and to their investments, as regards their operation in its territory, treatment no less favourable than the treatment it accords, in like situations, to its own investors and investments.	
Article X on Exclusions from investment liberalization chapter	
Articles X paragraph 1 (National Treatment), X (Most Favoured Nation Treatment), X (Performance Requirements), X (Senior Management and Board of Directors), do not apply to measures that a Party adopts or maintains with respect to sectors or subsectors as set out in its Annex II.	

The EU negotiation proposal for TTIP would have allowed investors in the United States to take the European Union Member States to investment arbitration and claim for compensation [38, 41], if investors/providers in the United States were put in worse competitive position in comparison to national investors/providers for health services. In the CETA this option was closed. In the CETA obligations on national treatment were not extended to services, which Member States had excluded. Our analysis indicate that the European Union did not deliver what it claimed to deliver and while health services were prioritised in comparison to other public services, establishment of new chapters has eroded scope of carve outs and undermined the MS reservations on investment and investment protection.

### The test on audiovisual services

New negotiation mandates are shaped by negotiation priorities of the European Union Member States. This is evident in the case of audiovisual services and France, which has been prominent in making a strict case for exclusion of audiovisual services as part of the EU negotiation mandate. It can thus be considered as a service category followed particularly closely by one Member State. If we compare health services to audiovisual services (such as motion picture services, tv and radio, sound recording) exclusion from EU trade negotiations, it is clear that audiovisual services are more extensively excluded than health services in CETA and in EU own TTIP services negotiation offer [36, 38]. European Union’s own TTIP services and investment chapter proposal includes simple exclusion of audiovisual services for electronic commerce and investment sections [38]. Similar exclusion could have been applied to health and social services more broadly, irrespective of how these are provided. However, it is important to note that while CETA excluded audiovisual services from investment liberalisation, audiovisual services were not excluded from investment protection [36]. This can imply that European Union priorities concerning investment protection were higher than interests of France or that France was willing to take the risk of becoming sued on the ground of investment protection obligations. Furthermore, text on not including audiovisual services was explicitly included in negotiation mandate of the CETA [42] and the TTIP [43]. While the scope for using “cultural exception” was more open in the CETA, the EU applied it only to audiovisual services and for the TTIP even this was more contested [44]. Audiovisual services were a specific concern for France, which actively pushed to exclude them already in discussions on negotiation mandate [45].

While there was scope to ensure that both health and audiovisual services were protected during negotiations, our results indicate that health services were not safeguarded as well as audiovisual services. However, both categories of services were included under investment protection. This suggests that trade negotiations are informed and may include concerns of Member States. It may also suggest that Member States should seek more explicit guidance for health services under negotiation mandate.

### The test on alternative means to achieve the same outcome: general obligations and principles in securing policy space

Our final test concerns alternative means to achieve the same outcome. In services negotiations three types of alternative avenues can be found, i) general exceptions, ii) broader goals and principles, e.g. right to regulate, iii) more explicit statements and declarations seeking to address the issue without changing the agreed text.

Trade agreements have always had exceptions, however, the preference of negotiators is to keep these narrow. This is the case for the “public services” or governmental services exception, which does not correspond to how modern publicly funded health systems operate as most health systems engage with outsourcing, public financing and other arrangements, where public services are in competition with private providers [37]. Pedreschi [24] has brought up how the European Union has balanced policy space by a piecemeal approach combining sectoral, general, and functional aspects. In addition to general exception clauses for public health and public morals, the European Union negotiated trade agreements include governmental services exclusion with reference to that of General Agreement on Trade in Services Article 1.3 [37]. However, this clause has been widely studied and it is now broadly accepted that it provides very limited exemption for publicly funded services [24, 46–48]. Arena has pointed out key issues as follows [46]:

‘*if governmental services under Article I:3(b) GATS are identified exclusively by reference to the two negative criteria in Article I:3(c) GATS, virtually all public services could be subject to the GATS, thus making the exemption meaningless’*.

In the case of CETA and TTIP negotiation mandates this is the only reference of services excluded from negotiations with no mention of health services and on health only for public health, such as references to occupational health and safety standards [42, 43].

The second group of alternative avenues are general statements, which emphasise “right to regulate”. Preambular statements before the actual agreement can express importance of an aspiration, e.g. for the right to regulate, but as they are not part of the agreement these remain aspirational. This is the case also for the CETA, which emphasises right to regulate in the preambular part of the agreement [36]:

*“RECOGNISING that the provisions of this Agreement preserve the right of the Parties to regulate within their territories and the Parties’ flexibility to achieve legitimate policy objectives, such as public health, safety, environment, public morals and the promotion and protection of cultural diversity”.*

A generic emphasis on right to regulate was added later to the investment protection chapter in CETA [36], but it merely reaffirms the right to regulate without relating this to breaching of obligations or claims of compensation in any other area than subsidies.

In the third group are separate declarations and clarifications. Trade negotiations are often accompanied by separate statements or letters, which seek to accommodate public concerns without impact on what is negotiated. A classic example is a letter to a government [49] or a statement on priorities [18]. These tend to follow articulation, which emphasizes that the agreement does not prevent from regulating, providing or subsidising services in general, but remain silent of the fact that the agreement could still impact on *how and on what ground a government will be able to regulate*. While such separate declarations and statements can give some support for interpretation, these are not part of the agreement and weaker than a negotiated text.

The use of separate statements, letters and declarations can be important for achieving consensus, but these are inappropriate means to address legitimate and valid public policy concerns. Van Harten [50] has, for example, brought up that the Joint Declaration on CETA did very little to address key concerns arising from privileges for foreign investors provided in CETA [51]. Similar separate provisions with respect to Member States statutory health care and social security systems can also be found, for example, in the EU internal market services directive [52].

Our results for the CETA and TTIP thus suggest that there are no effective broader exclusions, obligations, statements or requirements, which would cover health services concerns and provide an alternative avenue to achieving the same outcome in safeguarding regulatory policy space for health services.

## Discussion

A key dilemma and challenge for assessment of policy space arises from the pressure to narrow down what is included under “health services”. Services negotiated in other chapters may have major impact on health systems financing and provision. For example, e-commerce, health data and direct-to-consumer advertising can all be negotiated under different categories of services than health services. Health insurance services are also negotiated under financial services, where these may not be scrutinized as closely as under health services.

While we have focused mostly on services and investment chapters, health services could be affected by negotiations of several other chapters. For insurance-based health systems financial services chapter is important. Several chapters in new generation trade agreements, such as those on regulatory cooperation and principles, government procurement, state owned enterprises, technical barriers to trade, sanitary and phytosanitary measures, and competition can have further implications to health services governance and broader public health policies. Furthermore, it is likely that trade-related aspects of intellectual property will remain a key concern due to increasing prices of new medicines.

New chapters in trade agreements can provide new avenues for introduction of more general rules-based negotiations as specific exclusions apply to each chapter separately. Standardisation is discussed under trade agreements and could become more important regulatory instrument for trade in services [39, 53]. The comprehensiveness of the new generation trade agreements thus implies not only new openings (e.g. investment protection, regulatory cooperation, competition), but as well their more extensive and deeper reach to regulatory issues and local policies. New chapters can also be used to introduce measures, which become more important in the context of investment protection, such as procedural fairness or emphasis on regulatory principles. In 2016 the European Commission introduced a specific annex on medicinal products to regulatory cooperation and principles chapter proposal for TTIP [54].

The strongest case for positive impact in support of European Commission “walking the talk” is the European Union wide exclusion of health services with any public funding. Alternative explanations can be elaborated on the ground of MS pressure towards broader rather than narrower interpretation or a compromise following the move to negative listing for the CETA and the TTIP. Trade priorities tend to be defined by Ministries of Trade. Health services -related “defensive” requests may not be perceived as important or relevant from the perspective of Ministries of Trade in MS priority setting. However, irrespective of MS push the European Commission could have – as formally stated – prioritized health services also on European Union trade policy agenda. However, we do not see evidence of that and the comparison between the CETA and the TTIP point to the contrary.

European Union is the leading global trade block in the world. In principle, if protection of health and social services would have been important, European Union should have been able to prioritize excluding health services and statutory social security fully in its own trade negotiation proposals. An earlier trade policy document on Global Europe – competing in the world (2006) did not mention health, but emphasized that: “In terms of content new competitiveness-driven FTAs would need to be comprehensive and ambitious in coverage, aiming at the highest possible degree of trade liberalisation including far-reaching liberalisation of services and investment” [55].

Preference towards expanding trade agenda irrespective of consequences for health systems can be seen in the comparison between the CETA and the TTIP negotiation proposals. Investment protection chapter in the CETA reaffirms the right to regulate and only excludes forms of state aid explicitly [36]. The European Commission proposals for the TTIP were slightly different, but included additional emphasis on “necessary” measures [41]:

*“The provisions of this section shall not affect the right of the Parties to regulate within their territories through measures necessary to achieve legitimate policy objectives, such as the protection of public health, safety, environment or public morals, social or consumer protection or promotion and protection of cultural diversity*.”

The inclusion of “necessity” has been called as a “poison pill” in the right to regulate article [56]. This is because the word “necessary” defines policy measures. Necessity has a background in the WTO context as the necessity doctrine of “least trade restrictive” measures (see e.g. [51]). Investment arbitration has also addressed the issue strictly [51, 56]. Rather than establishing a right to regulate, the inclusion of necessity to further define it, in effect undermines and restricts the right to regulate to such regulation, which is the least trade restrictive. The reference to necessity was not included in the CETA, but it is in the European Union’s own offer for the TTIP, negotiation mandate for the CETA and amendment for negotiation on investment protection [41, 42, 57], which raises the question why the European Union independently sought to include it in trade agreements. The European Commission did not include an “umbrella clause” on government contracts to the CETA proposal on investment protection, while it was included in the European Union’s own TTIP offer to the United States. From a health policy perspective “umbrella” clause is a financial risk if a government wants to change contract terms or withdraw from outsourced services. Umbrella clauses have been considered problematic and their interpretation and use has varied between countries. Canada does not have umbrella clauses and France has these only in a minority of bilateral investment agreements [58]. Health systems have a substantial number of contractual relationships, which are potentially vulnerable to investment arbitration. For example, Poland [32] has been challenged to investment arbitration on the ground of an umbrella clause for retracting from privatisation.

A clear systemic point of concern remains for investment protection and willingness of the EU – and Member States - to include health services under investment protection. Investment protection sections include an article on transfers, which applies to the free movement of capital transfers and sets limits to how governments can interfere. Limitations to profits or other measures interfering with free transfer of capital could thus become a concern for publicly financed services. In the EU TTIP proposal, reservation has been made with respect to social security, public retirement or compulsory savings schemes [41]. Investment protection claim against Slovakia’s move towards non-profit basis was made on the ground of an article on transfers [31]. Threats of investment arbitration can be used to intimidate governments from addressing profiteering in publicly funded services or against moving back to public provision from outsourced services, even if this was compatible with obligations arising from the agreement otherwise. Investment protection needs to be considered also in relation to more systemic impacts on public policies, power and risk-sharing as emphasised by Koskenniemi [59].

The European Union as the leading global trade block in the world could have prioritized excluding health services fully in its own trade negotiation proposals, including from investment protection. The findings of our study support the necessity to focus on what is negotiated under so called free trade agreements as emphasised by Rodrik [60]. However, the European Commissions’ transparency with negotiation proposals has made this study possible and remains a key for research on what will be negotiated in future. While the European Commission may have been more responsive to health system concerns than many Member State governments, it has not done enough to match its own claims. This could have been easily done, for example, in EU’s own negotiation offers.

The European Union has been described as a conflicted power [61] or a benign power moving towards a social Europe reflecting French statist or more broadly social democratic emphases [62]. However, this does not seem to be in line with what the European Union has negotiated for trade. Our analysis is in line with Drieghe and Potjomkina [63] observations that irrespective of the values talk in trade policy, the aim of opening new markets has been pursued with great commitment even if it may not be in compliance with values promoted, requiring either a new narrative for trade policy or a change in negotiation priorities.

External policy discrepancy can reflect a similar trend in internal policies. The European Union external policies have become increasingly interlinked with internal policies during Juncker Commission, which had TTIP negotiations in a key role as a part the Commission work plan [64]. The European Union internal policies have been driven by negative integration [65, 66]. Conflicts between internal markets and health system values and principles were brought up already in 2006 in Council conclusions on common values and principles [67]. Member State concerns on availability, access and affordability of pharmaceuticals have also become explicit more lately [68]. However, European internal policies can be solved internally, whereas this is not the case for trade agreements.

## Conclusions

On the ground of the results from the three tests we conclude that the EU has not safeguarded health services in trade and investment negotiations, that it could have done it more extensively on the ground of outcomes with respect to audiovisual services, and that there are no indications that this could have been achieved through other means.

The European Union emphasis on exclusion of health services, which receive any public funding is a move to the right direction and in line with the policy stances, but it is not sufficient to guarantee the aims. This move is also accompanied by negative listing of services in new generation trade agreements, such as the CETA and the TTIP.

The negotiation outcomes in the CETA and the EU stances for TTIP negotiations imply that European Commission trade negotiations have sought to liberalise national treatment for all services as well as negotiated investment protection to cover all services and sectors, which do not comply with MS listing and EU stated priorities. These changes cannot be explained by inevitable compromises as result of tough negotiations or irrelevance as result of overarching obligations at the core of negotiations. More extensive exclusion of audiovisual services point out that strong priorities presented by prominent Member States can – to some extent – influence what is negotiated.

European trade and investment policy will need to comply with social Europe and public policy priorities of financial sustainability of health systems and statutory social security. This implies that commercial, investor, and corporate trade interests should not be prioritized over and above public interest and financial sustainability of health systems. This is a more systemic challenge between policy priorities for health and those for commercial policy both within the Member States and the European Union. European Union and the Member States have committed to ensuring health in all policies, economy of wellbeing and high level of health protection in all policies. Realising these commitments will require a fundamental change not only in processes, but priorities of trade and investment policies both in Europe and within Member States.

## Data Availability

All material used is publicly available and where necessary links have been provided in the manuscript. Data sharing is not applicable to this article as no datasets were generated or analysed during the current study.
